# Correction of sagittal imbalance in treatment for adult degenerative scoliosis with thoracic lordosis and lumbar kyphosis: A case report

**DOI:** 10.1097/MD.0000000000006416

**Published:** 2017-04-21

**Authors:** Tao Wang, Hui Wang, Lei Ma, Di Zhang, Wen-Yuan Ding

**Affiliations:** a Department of Spinal Surgery, The Third Hospital of Hebei Medical University; b Hebei Provincial Key Laboratory of Orthopedic Biomechanics, Shijiazhuang, China.

**Keywords:** adult degeneration scoliosis, lumbar kyphosis, from 5th thoracic to 12th thoracic lordosis

## Abstract

**Rationale:**

Lumbar degenerative scoliosis (LDS) is a common spinal disease for senior citizens. However, LDS accompanied with thoracic lordosis and lumbar kyphosis (LK) is rare in clinic. No reports have reported LDS with thoracic lordosis and LK.

**Patient concerns:**

A 54-year-old woman just complained about sever back pain without any radiculopathy and neurodeficit of low limb for 2 years, Visual Analogue Scale (VAS) for back pain was 9 points and x-ray showed adult LDS with lordosis angle of 10° from 5th thoracic to 12th thoracic (T5-T12) and LK angle of 20°.

**Diagnoses:**

She was diagnosed with adult degeneration scoliosis (ADS).

**Interventions:**

: The patient underwent posterior pedicle screw implantation from L1 to S1 levels.

**Outcomes:**

: Two weeks after surgery, VAS for back pain was 2 points and x-ray showed thoracic lordosis angle of 6°, lumbar lordosis (LL) of 6° and sagittal vertical axis from C7 plumb line (SVA) of 77 mm. One year after surgery, VAS for back pain was 1 points and the x-ray showed thoracic lordosis angle of 6°, LL of 20°, and SVA of 36 mm, implying globe spine for this patient tends to balance.

**Lessons:**

Adult degenerative scoliosis accompanied with thoracic lordosis and LK is rare. Correcting sagittal imbalance is an effective treatment. The surgical outcome is satisfactory. Attention should be paid in sagittal balance for treatment of ADS. We still need further follow-up to observe change of sagittal parameters.

## 1 Introduction

Adult degeneration scoliosis (ADS), a type of spinal scoliosis for elder adults without scoliosis history, is a complex spinal disease.^[1–3]^ The prevalence of ADS ranges from 1% to 10%.^[4]^ Spinal degeneration generally cause coronal scoliotic curvature after skeletal maturation and subsequent rotatory subluxation for multiple lumbar functional spinal units.^[3–6]^ With the advent of an aging society, the incidence of ADS increased.

Coronal Cobb angle, the rotation degree of the apical vertebra, and the position of the end and apical vertebrae are critical factors in the assessment of ADS.^[4,5]^ What is more, coronal Cobb angle is not only used for determining ADS classification but a crucial factor in selecting surgical approaches.^[7]^ However, recently it has been demonstrated that sagittal spinopelvic alignment plays an important role in determining the surgical selection. Faldini et al^[8]^ studied on how long or short fusions matter coronal and sagittal balance for adult lumbar degenerative scoliosis (LDS) and found that both can improve balance of the spine and correct Cobb angle in coronal plane. Sagittal balance must be considered in treatment for ADS. Here we showed a case of ADS with thoracic lordosis and lumbar kyphosis (LK). There is no report on ADS accompanied with thoracic lordosis and LK.

## 2 Consent

The current study was approved by ethics committee of the Third Hospital of Hebei Medical University. There is no need to obtain informed consent from the patient because all the data were collected and analyzed anonymously.

## 3 Case report

A 54-year-old adult woman complained about sever back pain without radiating symptom of lower limbs for 2 years. There is no other major medical history was noted. The patient performed on conservative treatment for 8 months, but it did not work. In physical examination, there was no cardiopulmonary deficits and the muscle power of the both low limb was 4/5 level. The Visual Analogue Scale (VAS) for back pain was 9 points. One years ago the patient checked positive and lateral full spine x-ray, magnetic resonance imaging (MRI) for lumbar and test for bone mineral density. x-Ray showed a lumbar scoliosis with a Cobb angle of 31°, a LK with a Cobb angle of 20° and from 5th thoracic to 12th thoracic lordosis (T5-T12 lordosis) with a Cobb angle of 10°, which showed Type L(31)HPC in Scoliosis Research Society (Fig. 1). MRI (Figs. 2 and 3) did not show any neurothlipsis and bone mineral density was normal. She was diagnosed with ADS.

**Figure 1:**
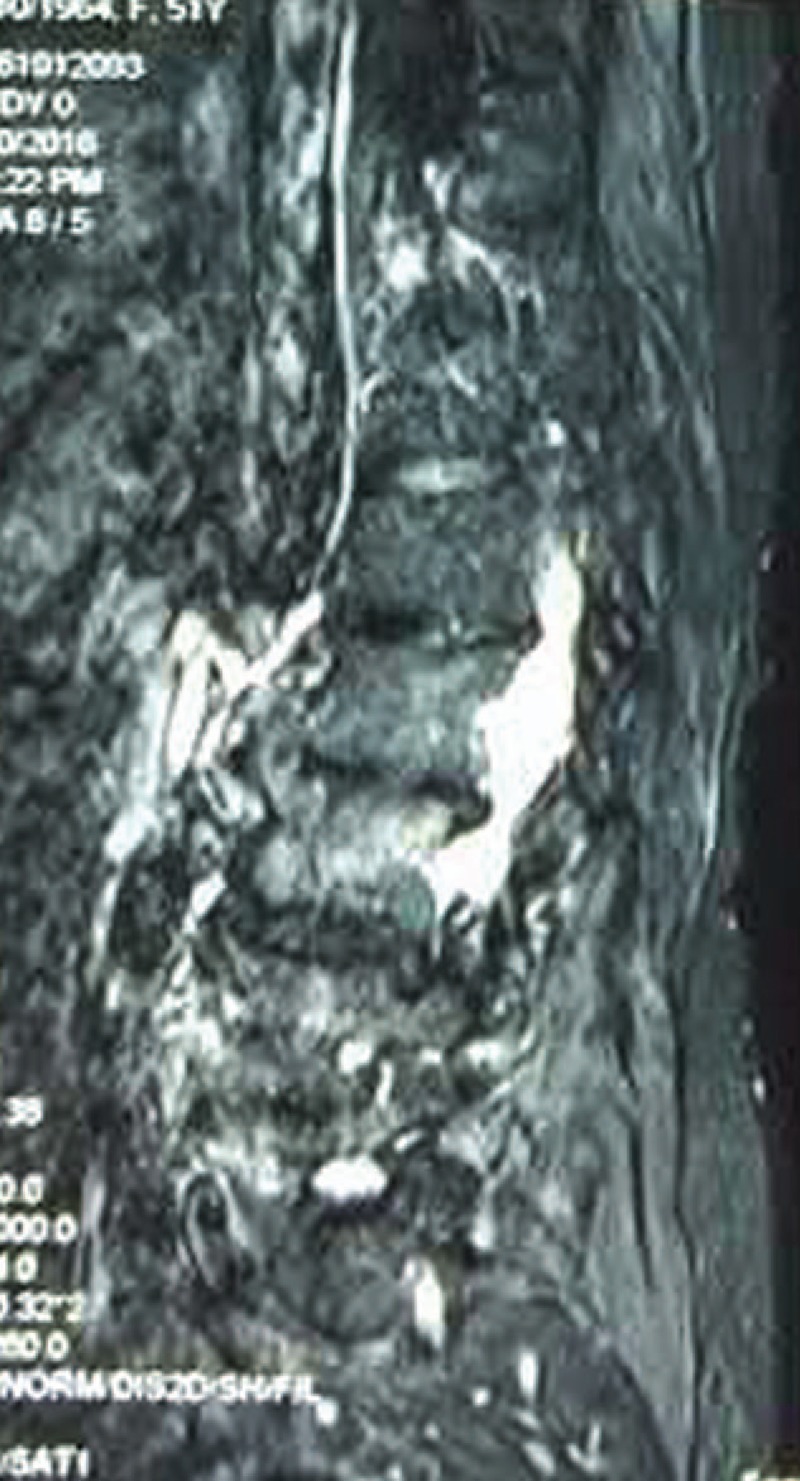
Positive and lateral full spine x-ray before surgery. PI = 60°, PT = 30°, SS = 30°, LL = −20°, T5-T12 = −10°, SVA = 75 mm. PI = pelvic incidence, PT = pelvic tilt, PT = pelvic tilt, SS = sacral slope, SVA = sagittal vertical axis from C7 plumb line.

**Figure 2:**
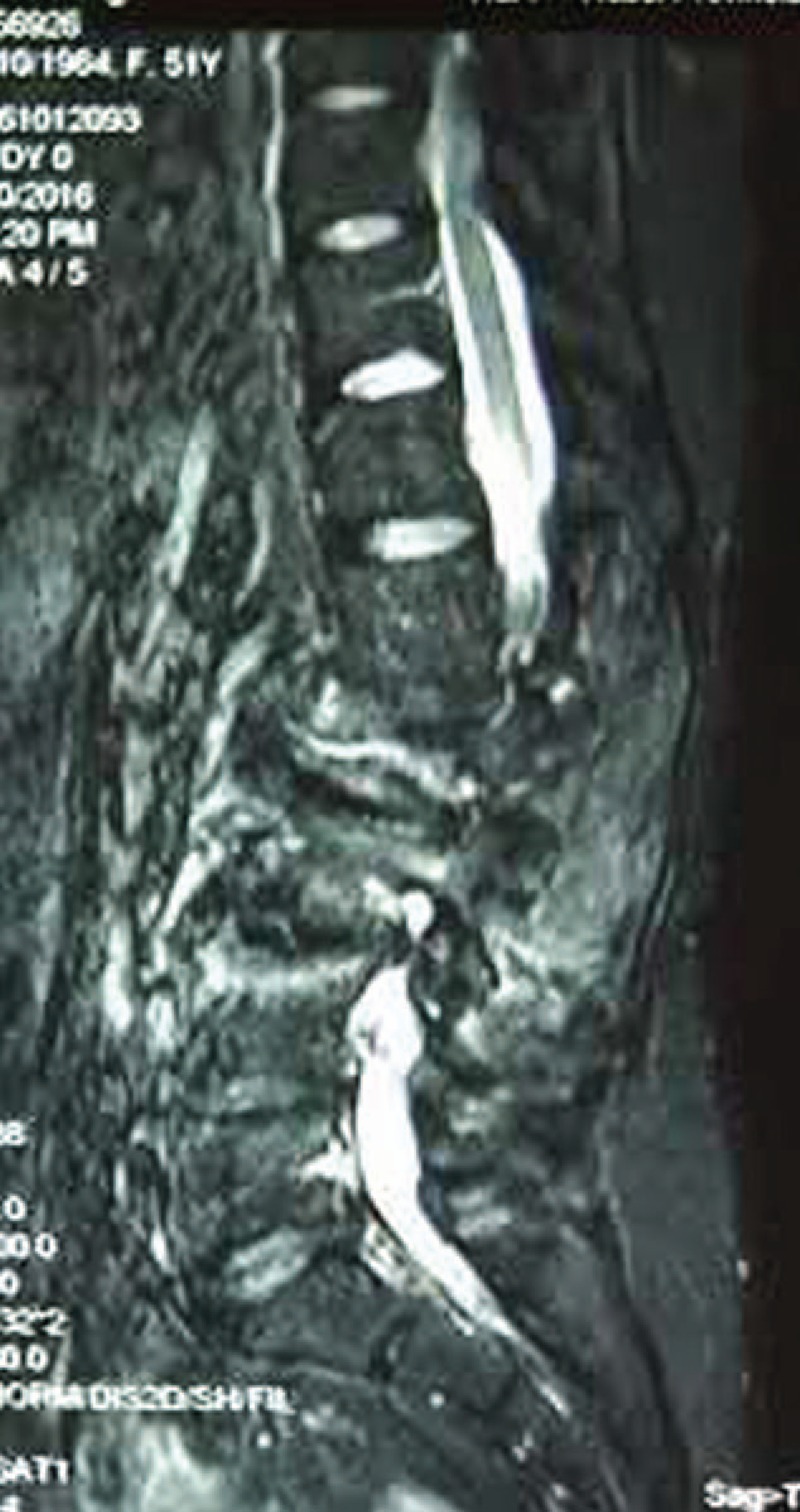
MRI for lumbar showed no neurothlipsis. MRI = magnetic resonance imaging.

**Figure 3:**
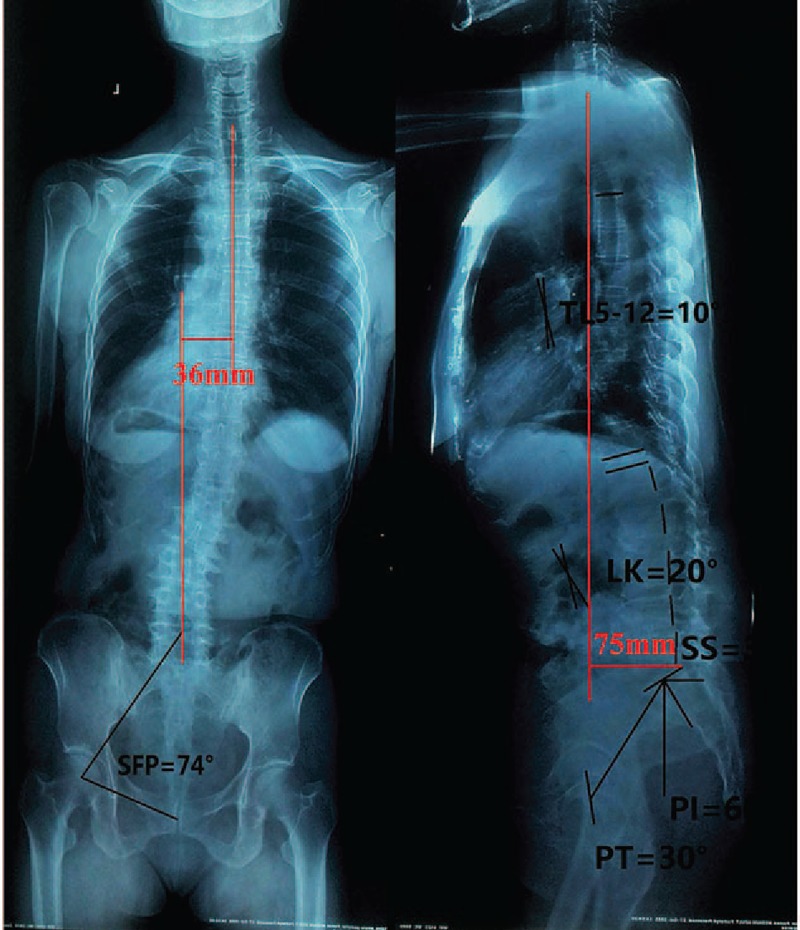
MRI for lumbar showed no neurothlipsis. MRI = magnetic resonance imaging.

After invalid conservative treatment and eliminating any explanation for sever back pain, we believed that the ADS was the “trigger point” of sever back pain. We chose corrected surgery at L1-S1 levels with pedicle screw. The patient felt back pain relief after surgery. Two weeks after surgery, the patient felt very well and VAS decreased from 9 points preoperatively to 2 points 2 weeks after surgery, indicating perfect efficacy of surgery. Cobb angles of lumbar scoliosis decreased from 31° to 0°, T5-T12 lordosis changed from 10° to 6 °and lumbar changed from 20° (kyphosis) to 6° (lordosis), as these showed in x-ray (Figs. 1 and 4). x-Ray also presented that pelvic incidence (PI) changed from 60° to 64°, pelvic tilt (PT) increased from 30° to 58°, sacral slope (SS) decreased from 30° to 6° and sagittal vertical axis from C7 plumb line (SVA) increased from 75 to 77 mm. One year after the operation, the patient felt much better (1 points for VAS) and the full spine x-ray showed that Cobb angles of lordosis lumbar increased from 6° to 20°, PI changed from 64°to 65°, PT decreased from 58° to 45°, SS increased from 6° to 20°, and SVA decreased from 77 to 36 mm (Figs. 1 and 5).

**Figure 4:**
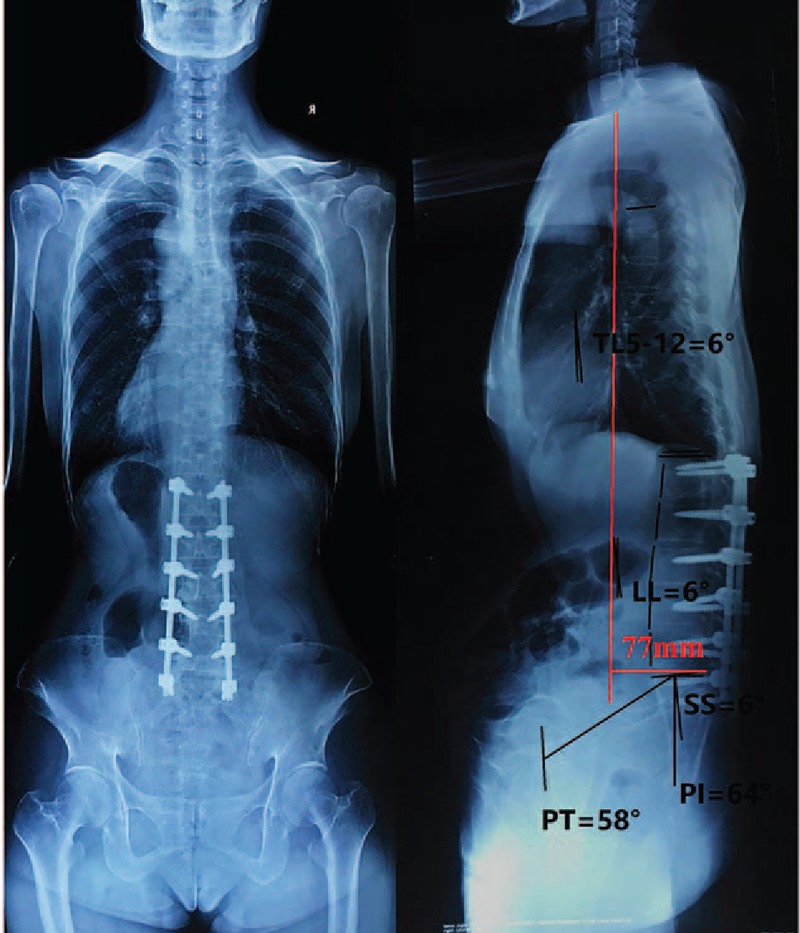
Positive and lateral full spine x-ray 2 weeks after surgery. PI = 64°, PT = 58°, SS = 6°, LL = 6°, T5-T12 = −6°, SVA = 77 mm. PI = pelvic incidence, PT = pelvic tilt, SS = sacral slope, SVA = sagittal vertical axis from C7 plumb line.

**Figure 5:**
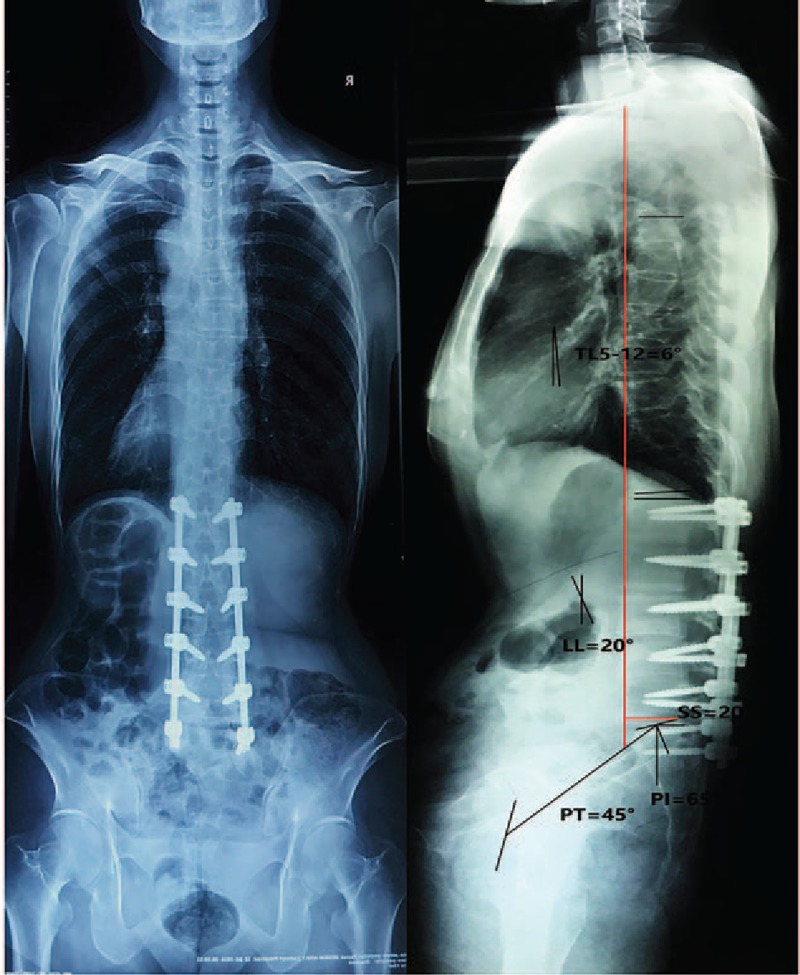
Positive and lateral full spine x-ray 1 year after surgery. PI = 65°, PT = 45°, SS = 20°, LL = 20°, T5-T12 = −6°, SVA = 36 mm. PI = pelvic incidence, PT = pelvic tilt, SS = sacral slope, SVA = sagittal vertical axis from C7 plumb line.

## 4 Discussion

In our case presents a 54-year-old woman with severe back pain without radiating symptom of lower limbs. x-Ray showed ADS with Cobb angle of 31° in coronal plane. This is a common disease in the elderly population. However, thoracic lordosis Cobb angle of 10° and LK Cobb angle of 20° was not very common. To the best of our knowledge, there was no study reporting this topic. Here, we present a case of thoracic lordosis and LK in a scoliosis patient.

Previously surgical option of ADS was based on Cobb angle in terms of coronal plane. Recent researches has shown that sagittal spinopelvic played a critical role alignment among patients with ADS.^[8–10]^ Schwab et al reported that PT = 22° or more, SVA = 47 mm or more, and PI − LL = 11° or more could estimate the severity of the deformity.^[9]^ Based on the points of Frank, the patient have sever spinal deformity due to PT = 30°, SVA = 75 mm, and PI − LL = 80° before surgery. After invalid conservative treatment and eliminating any explanation for sever back pain, we believed that ADS the key factors for sever back pain, so we just implanted pedicle screws at the level of L1-S1 with. We did not ignorance of sagittal imbalance, we just gave the spine an opportunity of self-correction depended on self-compensatory ability. After operation, ADS was corrected perfectly and T5-T12 lordosis corrected from 10° to 6° and LK 20° changed lordosis 6°. However, PT = 58°, SVA = 77 mm, and PI − LL = 58°, implying that this woman still had sever deformity 2 weeks after surgery. But 1 year after the surgery, the sagittal parameters had great changes, PT = 45°, SVA = 36 mm, and PI − LL = 45°, compared with these after surgery, indicating that the spine gradually tended to sagittal balance. Meanwhile, back pain for this patient markedly alleviated.

The mechanisms for this result are complex and unclear. We have a possible reason for explaining it. We observed that the center of gravity for the patient forward tilt, as shown in Fig. 1. However, after scoliosis correction surgery SS rapidly decreased causing center of gravity retroposition and pelvis forward and upward, as shown in Fig. 4. During 1 year, center of gravity retroposition made both lumbar compensatory lordosis and SS increase preventing further development of the spinal deformity. This is just a case and we need further follow-up to observe change of sagittal parameters.

In conclusion, LDS accompanied with thoracic lordosis and LK is rare in clinic. No reports have reported LDS with thoracic lordosis and LK. The current case suggests that sagittal imbalance of spine may be the key points for the patients with adult degenerative scoliosis who complains about serious back pain. Attention should be paid to sagittal balance for treatment of adult degenerative scoliosis. We provides a method for spinal surgeon when facing the rare case like this and we need further study to observe whether correction is loss or screw and rod are broken.
